# Multi-guild microbial cooperation sustains long-term anaerobic toluene degradation through sulfur cycling

**DOI:** 10.3389/fmicb.2026.1773863

**Published:** 2026-03-03

**Authors:** Bruna Matturro, Matteo Tucci, Andrea Firrincieli, Luca Niccolini, Verónica Peña-Álvarez, Marco Resitano, Martina Trinchillo, Ana Isabel Peláez, Simona Rossetti, Maurizio Petruccioli, Carolina Cruz Viggi, Federico Aulenta

**Affiliations:** 1 Water Research Institute (IRSA), National Research Council (CNR), Rome, Italy; 2 National Biodiversity Future Center, Palermo, Italy; 3 Department for Innovation in Biological, Agro-Food and Forest Systems (DIBAF), University of Tuscia, Viterbo, Italy; 4 Area of Microbiology, Department of Functional Biology and Environmental Biogeochemistry and Raw Materials Group, University of Oviedo, Oviedo, Spain; 5 Institute of Biotechnology of Asturias (IUBA), University of Oviedo, Oviedo, Spain

**Keywords:** anaerobic biodegradation, environmental microbiology, hydrocarbon-contaminated aquifers, metagenomics, sulfate-reducing bacteria, sulfur redox cycling, syntrophic consortia

## Abstract

Anaerobic degradation of aromatic hydrocarbons such as toluene plays a critical role in the natural and engineered attenuation of contaminated environments. Here, we developed and characterized a microbial consortium enriched under strictly anoxic conditions, capable of sustained toluene degradation through sulfate reduction. By integrating biodegradation kinetics, long-read 16S rRNA profiling, and genome-resolved metagenomics, we elucidated the structure and function of a multi-guild community. The consortium was co-dominated by *Desulfoprunum*, a sulfate-reducing bacterium (SRB), and *Sulfurovum*-affiliated sulfur oxidizers (~34% each), with additional members including *Stenotrophomonas, Achromobacter*, and *Stutzerimonas*. Such co-dominance appears uncommon, as sulfate-reducing enrichments are often characterized by low diversity and the predominance of a single lineage, such as *Desulfobacula* or *Desulfosarcina* in marine systems. Genome-resolved analyses recovered seven metagenome-assembled genomes (MAGs) with distinct but complementary metabolic roles. *Desulfoprunum* encoded the fumarate-addition pathway (*bss/bbs*) for anaerobic toluene activation and dissimilatory sulfate reduction (*aprAB, dsrAB*). In contrast, *Sulfurovum* and several *Gammaproteobacteria* encoded sulfide:quinone oxidoreductase (*sqr*), coupling H_2_S detoxification to energy conservation, while a *Moranbacterales* MAG carried a putative sulfhydrogenase (*hydAB*) potentially catalyzing elemental sulfur (S°) reduction. Additional MAGs encoded assimilatory sulfate reduction (*cys*), suggesting integration of sulfur into biosynthetic pathways. Together, these features are consistent with the presence of a putative distributed sulfur redox loop, in which biogenic H_2_S may be recycled via oxidation and reduction reactions mediated by co-occurring taxa. This sulfur loop is hypothesized to contribute to buffering sulfide toxicity and stabilize redox dynamics, thereby potentially supporting long-term toluene degradation under sulfidic conditions. Our findings highlight anaerobic degradation as a community-driven process enabled by sulfur-cycling interactions. By revealing the role of cryptic sulfur cycling in stabilizing hydrocarbon degradation, this work offers a new framework for designing bioremediation strategies in contaminated anoxic environments.

## Introduction

Petroleum hydrocarbons, including monoaromatic compounds like toluene, are pervasive contaminants in soils, sediments, and aquifers, arising from extraction, refining, storage, and accidental releases (Mekonnen and Aragaw, 2024). Among these, toluene is of particular concern due to its high solubility, mobility, and toxicity, which make it a frequent driver of groundwater contamination and particularly persistent in anoxic aquifers (Christensen et al., 2000; Zanello and Scherger, 2021). Understanding the mechanisms of toluene biodegradation is therefore central to predicting natural attenuation, designing remediation strategies, and advancing knowledge of subsurface microbial processes (Meckenstock et al., 2015; Tucci et al., 2022). While aerobic toluene degradation is well established, oxygen is rapidly depleted in contaminated plumes, shifting metabolism to anaerobic pathways (Beller et al., 1992; Lovley, 1995). Under oxygen-limited conditions, hydrocarbons can be degraded using alternative terminal electron acceptors, such as nitrate, ferric iron, sulfate, and carbon dioxide (Hernández-Ospina et al., 2024; Castro et al., 2022). Among these, sulfate is particularly relevant in both aquifers and marine settings (Widdel, 2001; Rabus et al., 2016). Sulfate-reducing bacteria (SRB) oxidize toluene via the fumarate addition pathway, catalyzed by benzylsuccinate synthase (BssA), with intermediates funneled through the benzoyl-CoA pathway before complete mineralization to CO_2_ (Beller et al., 1996). Sulfate is reduced to sulfide by enzymes such as ATP sulfurylase (Sat), adenosine-5′-phosphosulfate reductase (AprAB), and dissimilatory sulphite reductase (DsrAB). However, the concomitant production of sulfide can inhibit microbial activity and destabilize the degradation process (Beller, 1995).

Recent advances in microbial ecology have shown that sulfide does not necessarily accumulate but can be rapidly re-oxidized by sulfur-oxidizing bacteria, establishing a cryptic sulfur cycle, referred to as a cryptic sulfur loop, where reduced and oxidized sulfur species are rapidly cycled with minimal net accumulation (Canfield et al., 2010; Holmkvist and Ferdelman, 2011; Jørgensen and Findlay, 2019). While well documented in marine sediments and oxygen minimum zones, the relevance of such tightly coupled sulfur redox interactions in hydrocarbon-degrading consortia remains underexplored. Evidence suggests that anaerobic hydrocarbon degradation is not performed by SRB alone, but emerges from cooperative interactions among SRB, sulfur oxidizers, and facultative heterotrophs, which collectively stabilize metabolism under energy-limited conditions (Matturro and Ubaldi, 2016; Cruz Viggi et al., 2017; Wu et al., 2021).

In this study, we combined biodegradation kinetics, long-read 16S rRNA sequencing and metagenome-assembled genomes (MAGs) to investigate the microbial ecology of a toluene-degrading enrichment culture from a contaminated aquifer under sulfate-reducing conditions. This integrated approach allowed us to link kinetic performance with taxonomic structure and metabolic potential, uncovering a possible case of distributed cryptic sulfur cycle that contributes to fuel and stabilize hydrocarbon degradation. Indeed, our results reveal that toluene degradation under sulfate-reducing conditions is enabled by a cooperative microbial network that supports a cryptic sulfur cycle, contributing to the long-term stability of the process. By embedding toluene degradation within broader sulfur cycling networks, our findings extend the concept of cryptic sulfur cycling to anthropogenically impacted environments, with implications for both microbial ecology and applied bioremediation.

## Materials and methods

### Reagents and solutions

All chemicals used were of analytical grade and sourced from Merck KGaA (Darmstadt, Germany). De-ionized water (Millipore, Darmstadt, Germany) was utilized for preparing the mineral medium, the analytical standards, and all other solutions.

### Microbial culture

A bench-scale anaerobic bioreactor (total volume: 250 mL) was filled with 180 mL of mineral medium and inoculated with 20 mL of groundwater collected from a petrochemical-contaminated aquifer in Italy. The composition of the mineral medium is reported in Supplementary Table 1. Upon setup, the bioreactor was purged with pure nitrogen gas (N_2_) to establish anaerobic conditions. Toluene was the sole carbon and energy source and periodically spiked to reach a final concentration of 0.15 mM at the beginning of each feeding cycle. Each feeding cycle ended when toluene was almost completely degraded. Sulfate was also periodically added to a final concentration of 2 mM per cycle as the terminal electron acceptor. The culture was maintained under at room temperature (22 ± 3 °C) under continuous mixing, with pH kept constant at 7.2 ± 0.3.

### Sulfate reduction inhibition experiment

In order to confirm that the removal of toluene was coupled with the reduction of sulfate, microcosm experiments were set up in serum bottles (vol: 60 mL), containing 15mL of mineral medium and inoculated with 10mL of the microbial culture described in the previous paragraph after 150 days of enrichment. Oxygen was eliminated by N_2_ purging, and toluene and sulfate were added at a final concentration of 0.15mM and 2mmol/L respectively. Two kinds of treatments were implemented: in the first type, sodium molibdate was added (final concentration of 20Mm), while in the second (i.e., control) no molibdate was added. Molybdate is a well-known selective inhibitor reduction (Isa, 2004). The microcosms were kept under constant mixing by means of a magnetic stirrer and incubated at room temperature (22 ± 3 °C). Throughout the whole study, the pH was kept constant at 7.2 ± 0.3. Sulfate and toluene concentrations were monitored throughout the experiment.

### Chemical analyses

The presence of O_2_, H_2_, and CH_4_ was monitored by analyzing gaseous samples with a gas-chromatograph (Agilent 8860, GC system USA) equipped with a thermal conductivity detector (TCD). Toluene concentrations were measured by analyzing gaseous samples with a gas-chromatograph (Agilent 8860, GC system USA) equipped with a flame ionization detector (FID). Acetate concentrations were determined by injecting 1 μL of filtered (0.2 μm) and acidified (10 % vol/vol with 0.3 M oxalic acid) liquid samples, into a gas-chromatograph (Agilent 8860, GC system USA) equipped with a flame ionization detector (FID). The methods used for GC analysis, are reported in the Supporting Information (Tab. S2). Sulfate concentrations were determined by injecting a liquid sample (filtered, 0.22 μm porosity) into an ion chromatograph (IonPac AS14 analytical column, Dionex DX-100 system, Dionex Corp., Sunnyvale, CA, USA).

### DNA extraction and sequencing

Liquid sample (50 mL) of the culture were collected at steady state with a sterile syringe, filtered through hydrophilic polycarbonate membranes (0.2 μm pore size, 25 mm diameter, Millipore, Italy) and immediately processed for DNA extraction using the DNeasy PowerLyzer PowerSoil Kit (QIAGEN), following the manufacturer's protocol. DNA concentration (>100 ng/μL) and purity were assessed with the Qubit dsDNA HS Assay Kit and on the NanoDrop One device (both from Thermo Fisher Scientific, USA). DNA size distribution was analyzed using Genomic DNA ScreenTapes on the Agilent Tapestation 4200 (Agilent, USA). DNA was then used for long amplicon sequencing through 16S rRNA targeted and untargeted metagenomics. Amplicon libraries targeting the bacterial 16S rRNA gene were prepared using the Oxford Nanopore 16S Barcoding Kit (SQK-16S024, Oxford Nanopore Technology, ONT), according to the manufacturer's instructions. The genomic library was loaded onto a FLO-MIN106D flow cell (R9.4.1 chemistry) and sequenced using the MinION Mk1B device (Oxford Nanopore Technology, ONT). For shotgun metagenomics, barcoded SQK-NBD114.96 DNA libraries were prepared with minor modifications to the manufacturer's protocol (Oxford Nanopore Technologies, United Kingdom). The libraries were loaded onto primed FLO-PRO114M (R10.4.1) flow cells and sequenced on a PromethION P24 device running MinKNOW Release 24.02.19. Signal data was basecalled and demultiplexed using Dorado basecall server v. 7.3.11 (Oxford Nanopore Technologies) with the super-accurate (SUP) algorithm (dna_r10.4.1_e8.2_400bps_5khz_sup.cfg). Residual adapters and low-quality reads were trimmed using nanoq v. 0.10.0 with parameters -q 15, -l 1000, -S 150 and -E 150 to remove the first and last 150 bp of each read, along with any reads below q15 and length 1000 bp (Steinig, 2022).

The raw sequencing reads and metagenome-assembled genomes (MAGs) generated in this study have been deposited in NCBI under BioProject accession PRJNA1250843.

## Bioinformatics

### Nanopore long-read 16S rRNA amplicon sequencing and taxonomic profiling

Reads from 16S rRNA long amplicon sequencing were basecalled and demultiplexed using MinKNOW Guppy Dorado 7.3.9, employing the super-accurate basecalling algorithm (configurations: dna_r10.4.1_400bps_sup.cfg and dna_r9.4.1_450bps_sup.cfg). Bacterial 16S rRNA reads were filtered by length and quality using Chopper v0.8.0 (https://github.com/wdecoster/chopper), applying a minimum quality score of 9 and selecting reads between 1400 and 1700 bp. Taxonomic assignment and abundance calculations were performed using EMU v3.4.5 (Curry et al., 2022) with the Silva NR99 database v138.2.

### Metagenome assembly

Metagenome assemblies were generated using flye v. 2.9.4 with the parameters –meta and –extra-params min_read_cov_cutoff=5 (Kolmogorov et al., 2020). The assemblies were polished once using medaka v. 1.11.3 (https://github.com/nanoporetech/medaka) and contigs shorter than 1,000 bp were removed using seqkit v. 2.8.2 (Shen et al., 2016).

### MAG Binning and functional annotation

Metagenome-assembled genomes (MAGs) were binned with SemiBin v. 2.1.0 (–self-supervised, –sequencing-type long_read, –minfasta-kbs 500) using a self-supervised contrastive learning algorithm (Pan and Zhao, 2023). Inter-assembly MAG dereplication was performed using the drep v. 3.5.0 dereplicate workflow (-comp 50 -con 9.99 -p -l 500000) alongside checkm2 v. 1.0.2 (Olm et al., 2017; Chklovski et al., 2023). Dereplicated MAGs with a minimum completeness of 50% and contamination below 10% were functionally annotated with DRAM v.0.1.2 via KBase (Shaffer et al., 2020).

### MAG classification and phylogenomic analysis

The tool gtdbtk v. 2.4.0 was used to taxonomically classify MAGs against the Genome Taxonomy Database (GTDB) release 220 [https://doi.org/10.1093/bioinformatics/btz848]. Maximum likelihood phylogenomic tree was generated with GTDB-tk de-novo workflow through the identification, alignment, and concatenation of 120 phylogenetically informative marker genes (bac120). The resulting alignment file was finally analyzed in IQ-TREE (10.1093/molbev/msu300) to compute a maximum likelihood phylogenetic tree under the Whelan and Goldman (WAG) model with 1000 pseudo-bootstrap replicates (Aroney et al., 2025; Chaumeil et al., 2020; Nguyen et al., 2015).

## Calculations

The reaction describing the complete oxidation of toluene under sulfate-reducing conditions, not accounting for biomass growth, is reported below:


$$\begin{array}{l} 2C_{7}H_{8}+\;9{\;SO}_{4}^{2-}\to \;14\;{CO}_{2}+9\;S^{2-}+\;4H_{2}O \end{array}$$ (1)

To calculate the millimoles of electron equivalents (meq) generated by the complete oxidation of toluene to carbon dioxide, the following stoichiometric equation was used:


$$\begin{array}{l} C_{7}H_{8}+14H_{2}O\to \;7\;CO_{2}+\;36\;H^{+}+36\;e^{-} \end{array}$$ (2)

According to this equation, the factor used to convert the millimoles of toluene in milliequivalents is 36. Similarly, the millimoles of electron equivalents needed for sulfate reduction were calculated with a similar approach, using the following stoichiometric equations:


$$\begin{array}{l} SO_{4}^{2-}+8H^{+}+8\;e^{-}\to \;S^{2-}+\;4H_{2}O \end{array}$$ (3)

Accordingly, the factor used to convert the millimoles of sulfate in milliequivalents is 8.

## Results and Discussion

The integration of biodegradation kinetics with genome-resolved metagenomics provided insights into the kinetic, functional, and the ecological characteristics of a toluene-degrading enrichment culture under sulfate-reducing conditions. We firstly described the kinetic characterization of toluene degradation and sulfate reduction, followed by the analysis of microbial community composition and functional genomic potential. This combined approach allowed us not only to resolve the metabolic basis of the enrichment but also to link degradation performance to taxonomic structure and metabolic interactions. Finally, we highlight how ecological processes, in particular cryptic sulfur cycling, underpin hydrocarbon degradation and contribute to the long-term stability of the consortium due to the multi-guild bacterial cooperation, with broader implications for understanding the ecological role of sulfur cycling in hydrocarbon-impacted environments.

### Toluene degradation kinetics and sulfate reduction

**Figure 1:**
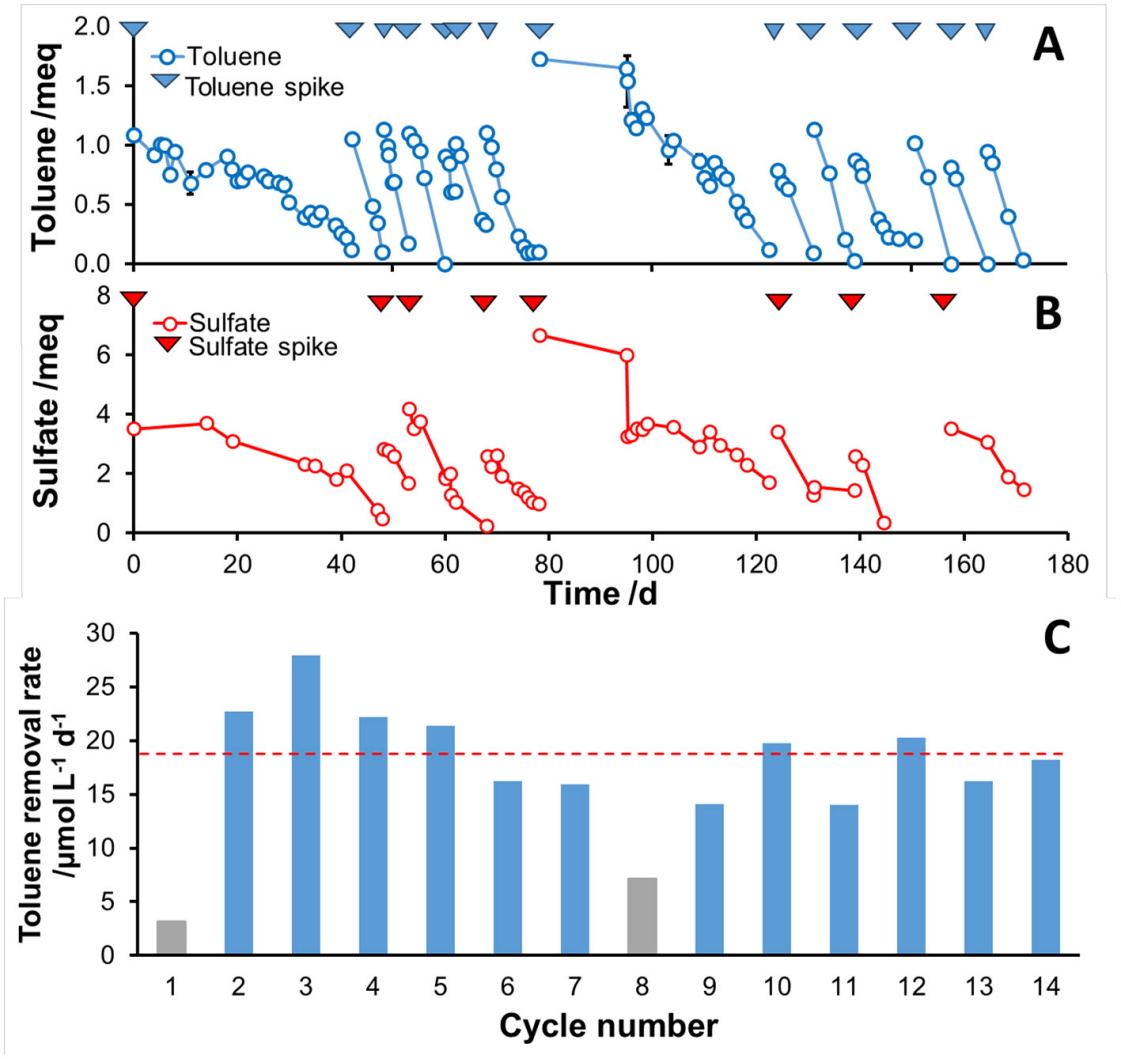
Temporal profiles of **(A)** toluene concentration and **(B)** sulfate concentration (expressed as milliequivalents). **(C)** Toluene removal rate per feeding cycle, expressed as μmol of toluene removed per day per unit liquid volume of the bioreactor. The average removal rate calculation excludes cycles 1 and 8, as the former represents the startup phase, while the latter was affected by toluene inhibition.

The temporal dynamics of toluene and sulfate concentrations over 170 days of operation are shown in Figure 1. After an initial startup phase (feeding cycle 1), toluene degradation rates increased significantly and remained stable in subsequent feeding cycles, indicating successful acclimation of the microbial consortium. Throughout the experiment, toluene degradation was consistently coupled to sulfate reduction, confirming the role of SO4^2−^ as the terminal electron acceptor for anaerobic toluene oxidation. During the 8th feeding cycle, the toluene and sulfate loads were doubled to test the robustness of the system. As a result, this perturbation caused a temporary decline in degradation rates, most likely due to toluene toxicity effects. However, from cycle 9 onward, when the initial substrate concentrations were restored, both toluene biodegradation and sulfate reduction fully recovered, returning to pre-inhibition levels. Excluding the startup phase (cycle 1) and the inhibited phase (cycle 8), the toluene removal rate remained stable, averaging 19.1 ± 1.2 μmol L^−1^ d^−1^ (Figure 1C). Throughout the entire experimental period, O_2_ was always below the detection limit, confirming strictly anaerobic conditions, and H_2_ and CH4 were not detected or remained at negligible levels. Additionally, acetate concentrations remained consistently below 5 mg L^−1^, indicating that it did not accumulate to a significant extent (data not shown).

From stoichiometric analysis of toluene consumption and sulfate reduction, we found that the observed cumulative sulfate consumption was approximately 1.9 ± 0.3 times higher than the theoretical value expected for complete oxidation of toluene to CO_2_ coupled to sulfate reduction. This imbalance can be attributed to “futile” redox cycles that dissipate the excess of reducing potential by consuming electron acceptors without contributing directly to substrate oxidation (Sharma and Khandelwal, 2024). It was recently reported that futile sulfur metabolism can be employed as strategy to achieve rapid sulfide detoxification while maintaining intracellular redox homeostasis under substrate-excess conditions (Jia et al., 2026).

To further confirm that the removal of toluene was coupled with the reduction of sulfate, we conducted a targeted experiment where molibdate was employed as selective inhibitor of sulfate reduction in a microcosm inoculated using the original enriched culture. As a result, negligible degradation was observed in a microcosm containing molibdate, while clear toluene removal occurred in the molibdate-free control (Supplementary Figure 1).

### Community composition and functional potential from MAGs

**Figure 2:**
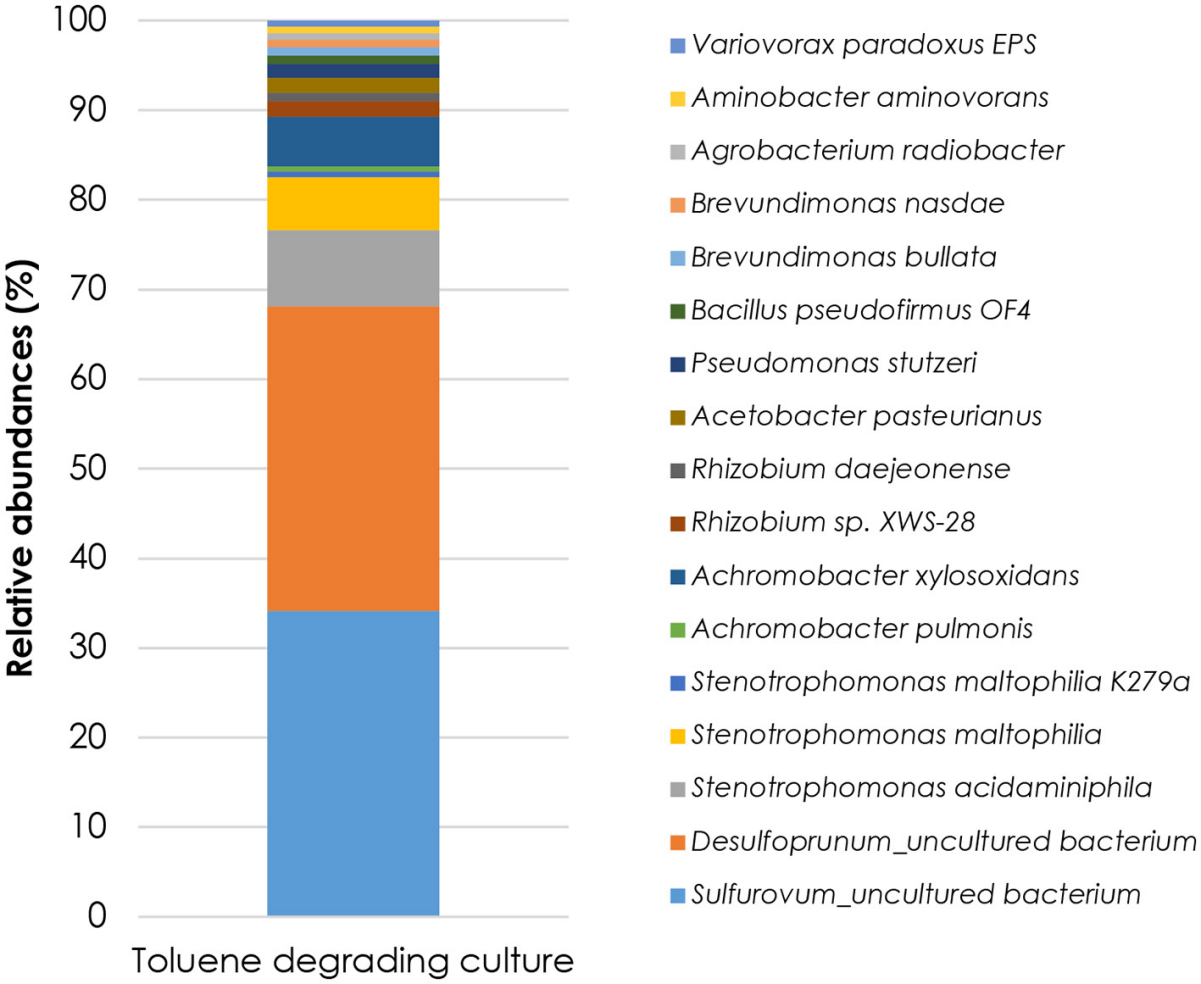
Microbial community composition of the enrichment culture as determined by16S rRNA long-reads sequencing.

Long-read 16S rRNA sequencing revealed a selected and low-complexity microbial consortium, co-dominated by *Desulfoprunum* (~34%) and *Sulfurovum*-affiliated sulfur oxidizers (*Sulfurovaceae*, ~34%), with minor contributions from *Stenotrophomonas* and *Achromobacter* spp. (< 10% each) and *Pseudomonas, Rhizobium, Bacillus, Variovorax*, and *Brevundimonas* (< 2%) (Figure 2). Such a co-dominance pattern is unusual, as sulfate-reducing enrichments are typically dominated by a single SRB lineage, such as *Desulfobacula* or *Desulfosarcina* in marine sediments, or *Desulfobulbaceae* in hydrocarbon-contaminated sites (Beller et al., 1996; Widdel, 2001; Kleinsteuber and Schleinitz, 2012; Rabus et al., 2016). Interestingly, the co-occurrence of SRB (*Desulfoprunum*), responsible for toluene degradation via dissimilatory sulfate reduction, and *Sulfurovum*-affiliated taxa, known to oxidize sulfide to sulfur, suggests a potential stabilizing mechanisms that may help mitigate the toxic effects of H_2_S, a byproduct of sulfate reduction, thereby supporting long-term toluene degradation.

**Figure 3:**
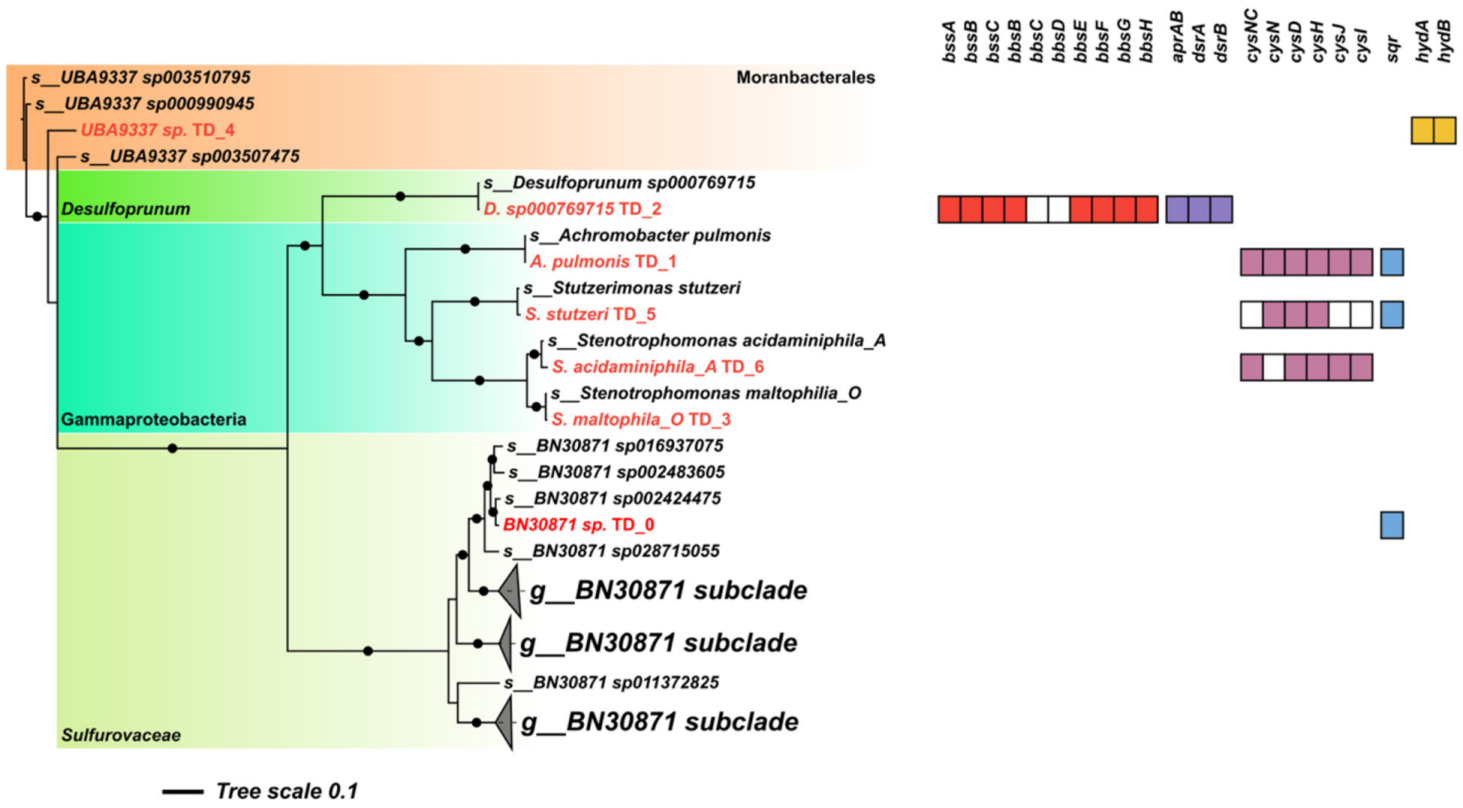
Phylogenomic placement of metagenome-assembled genomes (MAGs) recovered from the toluene-degrading enrichment culture, showing the distribution of key functional genes for anaerobic toluene degradation and sulfur cycling. The tree depicts the phylogenetic relationships among the reconstructed MAGs (phylogenomic tree), with black dots on nodes indicating bootstrap support (1,000 replicates) above 80%. The presence (color) or absence (white) of genes involved in the fumarate-addition pathway (bss/bbs) and sulfur metabolism is indicated. Functional annotations for each MAG are listed in Table S2.

Genome-centric metagenomic analysis using long-read sequencing supported this hypothesis, enabling the reconstruction of seven medium to high-quality MAGs (Supplementary Table 2): BN30871 sp. TD_0, *Achromobacter pulmonis* TD_1, *Desulfoprunum sp000769715* TD_2, UBA9337 sp. TD_4, *Stenotrophomonas acidaminiphila*_A TD_6, *Stenotrophomonas maltophila*_O TD_3, and *Stutzerimonas stutzeri* TD_5. Taxonomically, these MAGs are affiliated with the Patescibacteriota order *Moranbacterales* (UBA9337), Gammaproteobacteria (*S. acidaminiphila_A, S. maltophila_O, A. pulmonis, S. stutzeri*), *Desulfocapsaceae* (*Desulfoprunum sp000769715* TD_2), and *Sulfurovaceae* (BN30871) (Figure 3).

MAG-resolved analyses revealed that *Desulfoprunum sp000769715* TD_2 was the sole community member carrying the genes of the fumarate addition pathway (*bssABC, bbsEFGH*), which are required for the anaerobic activation of toluene and its conversion to benzoyl-CoA, together with the dissimilatory sulfate reduction genes (*aprAB, dsrAB*) (Winderl and Schaefer, 2007; Rabus et al., 2016). The genetic profile of *Desulfoprunum sp000769715* TD_2 is consistent with that of a canonical SRB capable of mineralizing aromatic hydrocarbons under anaerobic conditions. Indeed, previous studies have reported the involvement of *Desulfoprunum* species in anaerobic degradation of aromatic compounds under sulfate-reducing conditions, including toluene, further supporting its role as the key toluene degrader in this microbial consortium (Junghare, 2015; Irianni-Renno et al., 2024; Hudari, 2025).

**Figure 4:**
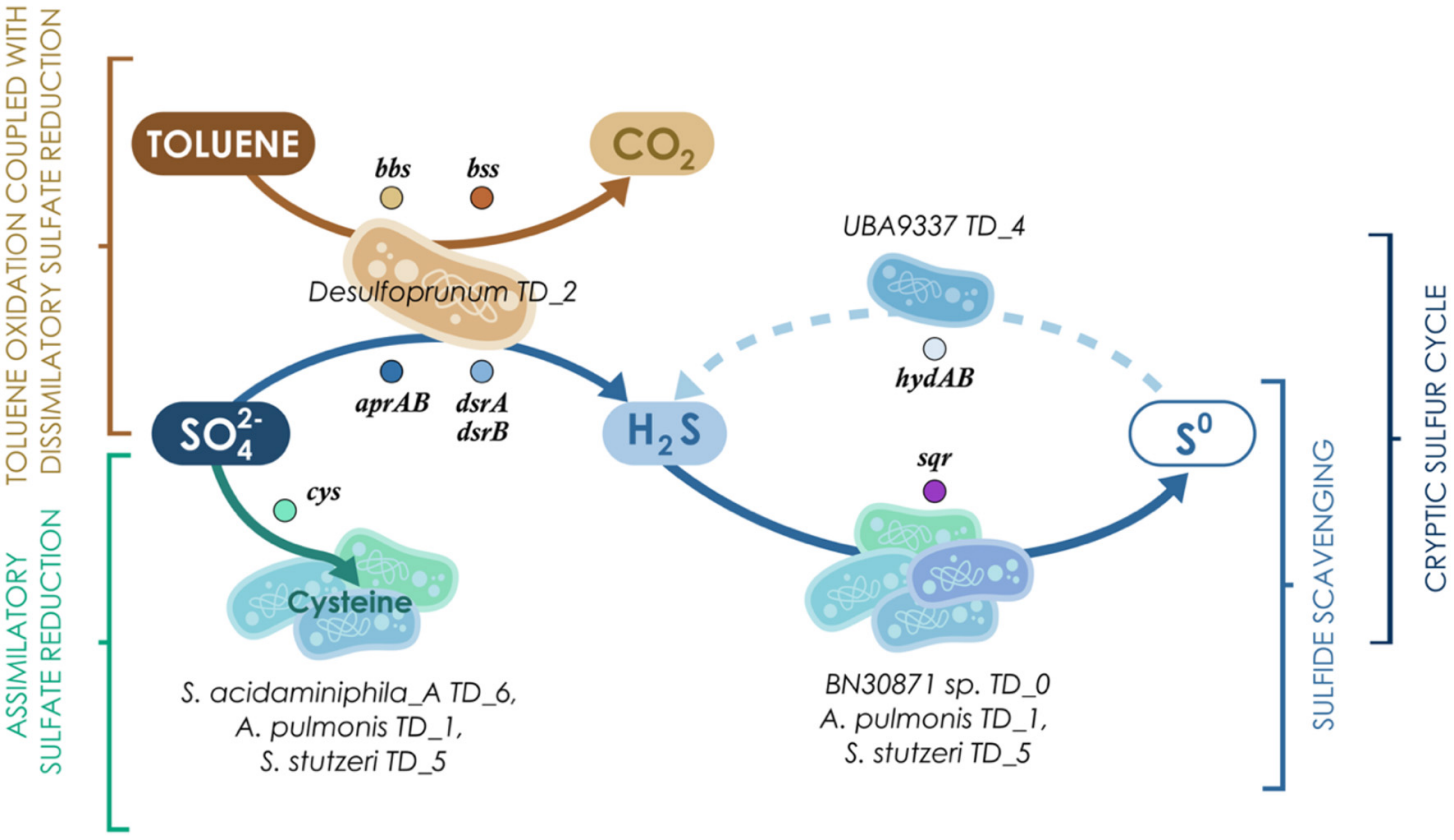
Proposed cryptic sulfur cycle in the toluene-degrading consortium. Schematic representation of MAG contributions to sulfur transformations, highlighting roles in dissimilatory and assimilatory sulfate reduction, sulfide oxidation, and sulfur reduction. Desulfoprunum sp000769715 TD_2 initiates anaerobic toluene degradation via the fumarate addition pathway (bssABC/bbsEFGH), converting toluene to benzoyl-CoA and CO_2_, with electrons transferred to sulfate, which is reduced to H_2_S through AprAB and DsrAB via dissimilatory sulfate reduction. The produced H_2_S is detoxified and oxidized to elemental sulfur (S°) by co-occurring taxa, including MAGs affiliated to Sulfurovaceae BN30871 sp. TD_0, Achromobacter pulmonis TD_1 and Stutzerimonas stutzeri TD_5, via sulfide:quinone oxidoreductase (sqr) with electrons feeding into the quinone pool of the electron transport chain. Elemental sulfur can then be reduced back to H_2_S by UBA9337 TD_4 (Moranbacterales) through a putative sulfhydrogenase (HydAB), using H_2_ as the electron donor. In parallel, Stenotrophomonas acidaminiphila_A TD_6, Achromobacter pulmonis TD_1, and Stutzerimonas stutzeri TD_5 perform assimilatory sulfate reduction through cys genes, channeling sulfate into cysteine biosynthesis for cellular metabolism. This combined network of dissimilatory, assimilatory, and recycling processes forms a cryptic sulfur loop that continuously regenerates oxidized and reduced sulfur species, mitigates sulfide toxicity, and sustains long-term sulfate reduction coupled to toluene oxidation. In the figure, genes encoding the proteins involved in the processes represented are indicated.

Interestingly, genes associated with other sulfur transformations, including assimilatory sulfate reduction (ASR), were more evenly distributed across the remaining MAGs (Figure 4). In particular, the Gammaproteobacteria MAGs (*A. pulmonis* TD_1, *S. stutzeri* TD_5, *S. acidaminiphila_A* TD_6) likely contributed to the sulfate demand, as they encoded nearly complete sets of *cys* genes required for assimilatory sulfate reduction to H_2_S (Kushkevych et al., 2020). In particular, *S. stutzeri* TD_5 harbors the complete set of *cysNCDHJI* genes required for assimilatory sulfate reduction (ASR) through the cysteine biosynthesis pathway (Figure 3). ASR is essential for the production of sulfur-containing amino acids such as cysteine and methionine. A key step shared with dissimilatory sulfate reduction (DSR) is the ATP-dependent activation of sulfate to adenosine-5′-phosphosulfate (APS), followed by its conversion to 3′-phosphoadenosine-5′-phosphosulfate (PAPS). PAPS is then reduced to sulfite and subsequently to sulfide by sulfite reductase, after which sulfide is incorporated into *L*-cysteine via O-acetyl-serine(thiol)-lyase. The synthesis of *L*-cysteine from inorganic sulfate thus represents the main route for sulfur incorporation into biomass under these conditions (Kushkevych et al., 2020). Additionally, *A. pulmonis* TD_1 and *S. stutzeri* TD_5, together with the *Sulfurovaceae* MAG (*BN30871* sp. TD_0), encode sulfide:quinone oxidoreductase (*sqr*), suggesting the potential for sulfide oxidation to elemental sulfur (S°), with electrons transferred to the quinone pool. While the presence of *sqr* indicates a pathway for sulfide detoxification, the ultimate electron acceptor in this anaerobic system remains unknown. Further studies are needed to clarify the electron transfer pathways involved in this process. This process links sulfide detoxification to energy conservation via reduction of the quinone pool (Landry and Ballou, 2021). Finally, the Patescibacteriota MAG *UBA9337* sp. TD_4 (*Moranbacterales*) encodes a putative sulfhydrogenase (HydAB), which may catalyze the reduction of elemental sulfur (S°) back to hydrogen sulfide (H_2_S) (Pedroni et al., 1995), thereby completing the cryptic sulfur cycle within the consortium.

Together, these complementary sulfur transformations outline a potentially distributed redox network that links toluene oxidation to internal sulfur turnover (Figure 4). In this network, DSR by *Desulfoprunum* couples' toluene oxidation with H_2_S release, while co-occurring taxa oxidize H_2_S to elemental sulfur (S°) via Sqr, thereby mitigating sulfide toxicity and regenerating electron acceptors. In parallel, other community members perform ASR to sustain biosynthetic demands, which likely accounts for the sulfate consumption (Figure 4).

Although similar sulfur cycling dynamics have been described in sedimentary systems, here we propose a conceptual framework in which tightly coupled sulfur redox interactions contribute to anaerobic hydrocarbon degradation in a microbial consortium. This framework is supported by genome-resolved evidence and community composition, rather than by direct measurements of intermediate sulfur species. In the enrichment studied here, toluene degradation does not appear to rely exclusively on canonical sulfate reduction but may be stabilized by a distributed sulfur-cycling network involving sulfate reducers, sulfide oxidizers, and sulfur assimilators (Figure 4).

Such coordinated interactions could enable internal sulfide detoxification and redox buffering, thereby sustaining long-term hydrocarbon degradation under sulfidic conditions.

From an ecological perspective, these findings reinforce the view of anaerobic hydrocarbon degradation as a community-driven process enabled by sulfur-cycling interactions, rather than the activity of a single taxonomic group. By integrating contaminant turnover with internal sulfur transformations, microbial consortia may enhance metabolic stability and resilience in anoxic environments.

Genome-resolved analyses reveal complementary metabolic roles across community members, providing a mechanistic basis for proposing cryptic sulfur cycling as a stabilizing feature of hydrocarbon-degrading communities. This model will benefit from future studies that directly quantify sulfur intermediates and functional activity (e.g., dissolved sulfide, elemental sulfur, proteomics), which will be important to confirm and refine our understanding of sulfur-coupled hydrocarbon degradation dynamics.

### Ecological interpretation and implications for bioremediation

The enrichment culture described here demonstrates how anaerobic toluene degradation can be sustained through cooperative sulfur cycling involving multiple microbial guilds. Within the consortium, *Desulfoprunum* performs toluene oxidation via the fumarate addition pathway (Bss/Bbs) coupled to dissimilatory sulfate reduction (AprAB/AsrAB), releasing H_2_S. This sulfide is subsequently detoxified and partially oxidized to elemental sulfur (S°) by co-occurring taxa, including *Sulfurovaceae, Achromobacter, Stenotrophomonas*, and *Stutzerimonas* carrying the *sqr* gene, while UBA9337 (*Moranbacterales*), encoding HydAB, may reduce S° back to H_2_S (Figure 4), potentially closing a cryptic sulfur cycling. This distributed sulfur transformation network likely acts as a redox-buffering and detoxification mechanism, preventing sulfide accumulation. By partitioning key sulfur processes across specialized taxa, the community maintains redox balance and minimizes sulfide inhibition. Although our enrichment culture lacks the large-scale spatial stratification observed in natural environments (e.g., marine sediments or oxygen minimum zones), the co-occurrence of sulfate-reducing and putative sulfur-oxidizing taxa, together with their respective functional genes (e.g., *dsrAB, aprAB, sqr, hydAB*), suggests potential metabolic complementarity. This raises the hypothesis that microscale redox heterogeneity, possibly arising within microbial aggregates or biofilms, may enable the partial coexistence of these energetically opposing sulfur transformations even in a well-mixed bioreactor system (Jia et al., 2026). Nonetheless, we emphasize that this model is based on genome-resolved inference and future work will be needed to validate these interactions, including direct measurements of sulfur species and spatially resolved analyses to confirm the presence of redox gradients and the activity of the implicated pathways.

From an applied perspective, this cooperative sulfur metabolism offers a mechanistic explanation for the functional resilience and robustness of anaerobic hydrocarbon-degrading consortium. Such internal sulfur cycling could enhance the performance of *in situ* bioremediation systems, including engineered bioreactors, by mitigating sulfide toxicity and sustaining electron flow, provided that sulfate remains available.

Ecologically, these findings extend the relevance of cryptic sulfur cycling, previously described in marine sediments, to hydrocarbon-fed consortia in contaminated aquifers. In this context, hydrocarbons appear to fuel a distributed sulfur redox network involving reducers, oxidizers, and recyclers. Such multi-guild cooperation may stabilize degradation processes, enhances community resilience under stress conditions (e.g., contamination), and embed contaminant turnover within broader carbon–sulfur biogeochemical coupling. If widespread, hydrocarbon-driven cryptic sulfur cycling could represent a general ecological mechanism that sustains microbial functioning and contaminant attenuation in anthropogenically impacted anoxic environments.

## Conclusions

This study offers a conceptual framework for understanding how sulfur-cycling interactions can support anaerobic hydrocarbon degradation. Our findings show that anaerobic hydrocarbon degradation in sulfidic environments may arise from cooperative interactions among multiple microbial guilds linked through cryptic sulfur cycling. This distributed redox network has the potential to mitigate sulfide toxicity, buffer environmental fluctuations, and underpins the long-term stability of degradation. Future work should aim to disentangle the specific metabolic roles of individual taxa, resolve interspecies electron transfer mechanisms, and assess the prevalence of sulfur-mediated stabilization in natural and engineered systems. Beyond the enrichment-scale context, these dynamics reveal broader principles of carbon–sulfur coupling in anthropogenically impacted subsurface environments and offer a conceptual basis for designing bioremediation strategies that harness multi-guild microbial cooperation.

## Data Availability

The data presented in this study are publicly available. The data can be found here: https://www.ncbi.nlm.nih.gov/bioproject, accession PRJNA1250843.

## References

[B1] Aroney S. T. N. Newell R. J. P. Nissen J. N. Camargo A. P. Tyson G. W. Woodcroft B. J. (2025). CoverM: read alignment statistics for metagenomics. Bioinformatics 41:btaf147. doi: 10.1093/bioinformatics/btaf147 40193404 PMC11993303

[B2] Beller H. R. (1995). The role of iron in enhancing anaerobic toluene degradation in sulfate-reducing enrichment cultures. Microb. Ecol. 30, 105–114. doi: 10.1007/BF00184517 24185416

[B3] Beller H. R. Grbić-Galić D. Reinhard M. (1992). Microbial degradation of toluene under sulfate-reducing conditions and the influence of iron on the process. Appl. Environ. Microbiol. 58, 786–793. doi: 10.1128/aem.58.3.786-793.1992 1575481 PMC195335

[B4] Beller H. R. Spormann A. M. Sharma P. K. Cole J. R. Reinhard M. (1996). Isolation and characterization of a novel toluene-degrading, sulfate-reducing bacterium. Appl. Environ. Microbiol. 62, 1188–1196. doi: 10.1128/aem.62.4.1188-1196.1996 8919780 PMC167885

[B5] Canfield D. E. Stewart F. J. Thamdrup B. De Brabandere L. Dalsgaard T. Delong E. F. . (2010). A cryptic sulfur cycle in oxygen-minimum-zone waters off the Chilean Coast. Science 330, 1375–1378. doi: 10.1126/science.1196889 21071631

[B6] Castro A. R. Martins G. Salvador A. F. Cavaleiro A. J. (2022). Iron compounds in anaerobic degradation of petroleum hydrocarbons: a review. Microorganisms 10:2142. doi: 10.3390/microorganisms10112142 36363734 PMC9695802

[B7] Chaumeil P.-A. Mussig A. J. Hugenholtz P. Parks D. H. (2020). GTDB-Tk: a toolkit to classify genomes with the Genome Taxonomy Database. Bioinformatics 36, 1925–1927. doi: 10.1093/bioinformatics/btz848 31730192 PMC7703759

[B8] Chklovski A. Parks D. H. Woodcroft B. J. Tyson G. W. (2023). CheckM2: a rapid, scalable and accurate tool for assessing microbial genome quality using machine learning. Nat Methods 20, 1203–1212. doi: 10.1038/s41592-023-01940-w 37500759

[B9] Christensen T. H. Bjerg P. L. Banwart S. A. Jakobsen R. Heron G. Albrechtsen H-J. (2000). Characterization of redox conditions in groundwater contaminant plumes. J. Cont. Hydrol. 45, 165–241. doi: 10.1016/S0169-7722(00)00109-1

[B10] Curry K. D. Wang Q. Nute M. G. Tyshaieva A. Reeves E. Soriano S. . (2022). Emu: species-level microbial community profiling of full-length 16S rRNA Oxford Nanopore sequencing data. Nat Methods 19, 845–853. doi: 10.1038/s41592-022-01520-4 35773532 PMC9939874

[B11] Hernández-Ospina D. A. Osorio-González C. S. Miri S. . (2024). New perspectives on the anaerobic degradation of BTEX: mechanisms, pathways, and intermediates. Chemosphere 361:142490. doi: 10.1016/j.chemosphere.2024.142490 38821131

[B12] Holmkvist L. Ferdelman T. G. (2011). A cryptic sulfur cycle driven by iron in the methane zone of marine sediment (Aarhus Bay, Denmark). Geochimica et Cosmochimica Acta 75, 3581–3599. doi: 10.1016/j.gca.2011.03.033

[B13] Hudari M. S. B. (2025). Sulfidic toluene mineralization by aquifer microbial communities at different temperatures. FEMS Microbiol. Ecol. 101:fiaf079. doi: 10.1093/femsec/fiaf079 40728916 PMC12342453

[B14] Irianni-Renno M. Rico J. L. Key T. A. De Long S. K. (2024). Evaluating natural source zone depletion and enhanced source zone depletion in laboratory columns via soil redox continuous sensing and microbiome characterization. J. Hazard. Materials 477135059. doi: 10.1016/j.jhazmat.2024.135059 39053064

[B15] Isa M. H. (2004). (2005). Molybdate inhibition of sulphate reduction in two-phase anaerobic digestion. Process Biochem. 40, 2079–2089, 025. doi: 10.1016/j.procbio.2004.07.025

[B16] Jia T. Peng Y. Niu L. Qi Z. (2026). Simultaneous sulfide oxidation and sulfate reduction for intracellular redox homeostasis under highly acidic conditions. Nat Commun. 17:1797. doi: 10.1038/s41467-026-68508-y 41554726 PMC12917267

[B17] Jørgensen B. B. Findlay A. J. (2019). The biogeochemical sulfur cycle of marine sediments. Front. Microbiol. 10:00849, 00849. doi: 10.3389/fmicb.2019.00849 31105660 PMC6492693

[B18] Junghare M. (2015). Desulfoprunum benzoelyticum gen. nov., sp. nov., a Gram-negative, benzoate-degrading, sulfate-reducing bacterium isolated from a wastewater treatment plant. Int. J. Syst. Evol. Microbiol. 65, 77–84. 066761–0. doi: 10.1099/ijs.0.066761-0 25278560

[B19] Kleinsteuber S. Schleinitz K. M. (2012). Key players and team play: anaerobic microbial communities in hydrocarbon-contaminated aquifers. Appl. Microbiol. Biotechnol. 94, 851–873. doi: 10.1007/s00253-012-4025-0 22476263

[B20] Kolmogorov M. Bickhart D. M. Behsaz B. Gurevich A. Rayko M. Shin S. B. . (2020). metaFlye: scalable long-read metagenome assembly using repeat graphs. Nat Methods, 17, 1103–1110. doi: 10.1038/s41592-020-00971-x 33020656 PMC10699202

[B21] Kushkevych I. Cejnar J. Treml J. Dordević D. Kollar P. Vítězová M. (2020). Recent advances in metabolic pathways of sulfate reduction in intestinal bacteria. Cells 9:698. doi: 10.3390/cells9030698 32178484 PMC7140700

[B22] Landry A. P. Ballou D. P. (2021). Hydrogen sulfide oxidation by sulfide quinone oxidoreductase. ChemBioChem 22, 949–960. doi: 10.1002/cbic.202000661 33080111 PMC7969369

[B23] Lovley D. R. (1995). Deep subsurface microbial processes. Rev. Geophys. 33, 365–381. doi: 10.1029/95RG01305

[B24] Matturro B. Ubaldi C. (2016). Microbiome Dynamics of a Polychlorobiphenyl (PCB) historically contaminated marine sediment under conditions promoting reductive dechlorination. Front. Microbiol. 7:1502. doi: 10.3389/fmicb.2016.01502 27708637 PMC5030254

[B25] Meckenstock R. U. Elsner M. Griebler C. Lueders T. Stumpp C. Aamand J. . (2015). Biodegradation: updating the concepts of control for microbial cleanup in contaminated aquifers. Environ. Sci. Technol. 49, 7073–7081. doi: 10.1021/acs.est.5b00715 26000605

[B26] Mekonnen B. A. Aragaw T. A. (2024). Bioremediation of petroleum hydrocarbon contaminated soil: a review on principles, degradation mechanisms, and advancements. Front. Environ. Sci. 12:1354422. doi: 10.3389/fenvs.2024.1354422

[B27] Nguyen L-T. Schmidt H. A. Von Haeseler A. (2015). IQ-TREE: a fast and effective stochastic algorithm for estimating maximum-likelihood phylogenies. Mol. Biol. Evol. 32, 268–274. doi: 10.1093/molbev/msu300 25371430 PMC4271533

[B28] Olm M. R. Brown C. T. Brooks B. Banfield J. F. (2017). dRep: a tool for fast and accurate genomic comparisons that enables improved genome recovery from metagenomes through de-replication. ISME J. 11, 2864–2868. doi: 10.1038/ismej.2017.126 28742071 PMC5702732

[B29] Pan S. Zhao X-M. (2023). SemiBin2: self-supervised contrastive learning leads to better MAGs for short- and long-read sequencing. Bioinformatics 39, i21–i29. doi: 10.1093/bioinformatics/btad209 37387171 PMC10311329

[B30] Pedroni P. Volpe A. D. Galli G. Mura G. M. Pratesi C. Grandi G. (1995). Characterization of the locus encoding the [Ni-Fe] sulfhydrogenase from the archaeon Pyrococcus furiosus: evidence for a relationship to bacterial sulfite reductases. Microbiology 141, 449–458. doi: 10.1099/13500872-141-2-449 7704275

[B31] Rabus R. Boll M. Heider J. Meckenstock R. U. Buckel W. Einsle O. . (2016). Anaerobic microbial degradation of hydrocarbons: from enzymatic reactions to the environment. J. Mol. Microbiol. Biotechnol. 26, 5–28. doi: 10.1159/000443997 26960061

[B32] Shaffer M. Borton M. A. McGivern B. B. Zayed A. A. La Rosa S. L. Solden L. M. . (2020). DRAM for distilling microbial metabolism to automate the curation of microbiome function. Nucleic Acids Res. 48, 8883–8900. doi: 10.1093/nar/gkaa621 32766782 PMC7498326

[B33] Sharma A. K. Khandelwal R. (2024). Futile cycles: emerging utility from apparent futility. Cell Metab. 36, 1184–1203. doi: 10.1016/j.cmet.2024.03.008 38565147

[B34] Shen W. Le S. Li Y. Hu F. (2016). SeqKit: a cross-platform and ultrafast toolkit for FASTA/Q file manipulation. PLoS ONE 11:e0163962. doi: 10.1371/journal.pone.0163962 27706213 PMC5051824

[B35] Steinig E. (2022). Nanoq: ultra-fast quality control for nanopore reads. J. Open Source Softw. 7:2991. doi: 10.21105/joss.02991

[B36] Tucci M. Viggi C. C. Crognale S. Matturro B. Rossetti S. Capriotti A. L. . (2022). Insights into the syntrophic microbial electrochemical oxidation of toluene: a combined chemical, electrochemical, taxonomical, functional gene-based, and metaproteomic approach. Sci. Total Environ. 850:157919. doi: 10.1016/j.scitotenv.2022.157919 35964739

[B37] Viggi C. C. Matturro B. Frascadore E. Insogna S. Mezzi S. Kaciulis S. . (2017). Bridging spatially segregated redox zones with a microbial electrochemical snorkel triggers biogeochemical cycles in oil-contaminated River Tyne (UK) sediments. Water Res. 127, 11–21. doi: 10.1016/j.watres.2017.10.002 29020640

[B38] Widdel F. (2001). Anaerobic biodegradation of saturated and aromatic hydrocarbons. Curr. Opin. Biotechnol. 12, 259–276. doi: 10.1016/S0958-1669(00)00209-3 11404104

[B39] Winderl C. Schaefer S. (2007). Detection of anaerobic toluene and hydrocarbon degraders in contaminated aquifers using benzylsuccinate synthase (bssA) genes as a functional marker. Environ. Microbiol. 9, 1035–1046. doi: 10.1111/j.1462-2920.2006.01230.x 17359274

[B40] Wu B. Liu F. Fang W. Yang T. Chen G-H. He Z. . (2021). Microbial sulfur metabolism and environmental implications. Sci. Total Environ. 778:146085. doi: 10.1016/j.scitotenv.2021.146085 33714092

[B41] Zanello V. Scherger L. E. (2021). Assessment of groundwater contamination risk by BTEX from residual fuel soil phase. SN Appl. Sci. 3:307. doi: 10.1007/s42452-021-04325-w

