# Supplementary material for: Multi-guild microbial cooperation sustains long-term anaerobic toluene degradation through sulfur cycling
Source: Front Microbiol. 2026 Mar 3;17:1773863. doi: 10.3389/fmicb.2026.1773863 (PMC12992304; doi:10.3389/fmicb.2026.1773863)
Supplement: Supplementary file 1 [file Data_Sheet_1.pdf]

Supplemental Material

Multi-guild microbial cooperation sustains long-term anaerobic toluene degradation through sulfur cycling

Bruna Matturro[1,2*], Matteo Tucci[1], Andrea Firrincieli[3], Luca Niccolini[1], Verónica Peña-Álvarez[4], Marco Resitano[1], Martina Trinchillo[1], Ana Isabel Peláez[4], Simona Rossetti[1], Maurizio Petruccioli[3], Carolina Cruz Viggi[1], Federico Aulenta[1,2]

[1] *Water Research Institute (IRSA), National Research Council (CNR), 00010 Montelibretti (RM), Italy*

[2] *National Biodiversity Future Center, Palermo, 90133, Italy*

[3] *Department for Innovation in Biological, Agro-Food and Forest Systems (DIBAF), University of Tuscia, Viterbo, 01100, Italy*

[4]*Area of Microbiology, Department of Functional Biology and Environmental Biogeochemistry and Raw Materials Group, University of Oviedo, Spain; Institute of Biotechnology of Asturias (IUBA), University of Oviedo, Spain.*

**Authors contributed equally*

Corresponding author: bruna.matturro@irsa.cnr.it

Table S1 – Composition of the mineral medium

**Minerals**	
**Compound**	**Concentration (g/L)**
NH_4Cl	0.5
$MgCl_2 \times 6H_2O$	0.1
$CaCl_2 \times 2H_2O$	0.05
K_2HPO_4	0.4
**Trace metals**	
**Compound**	**Concentration (mg/L)**
Nitrilotriacetic acid	4.5
$FeSO_4 \times 7H_2O$	0.556
$MnSO_4 \times H_2O$	0.086
$CoCl_2 \times 6H_2O$	0.17
$ZnSO_4 \times 7H_2O$	0.21
H_3BO_3	0.019
$NiCl_2$	0.02
Na_2MoO_4	0.01
**Vitamins**	
**Compound**	**Concentration (mg/L)**
Biotin (B7)	0.02
Folic acid (B9)	0.02
Pyridoxine (B6)	0.1
Thiamine (B1)	0.05
Riboflavin (B2)	0.05
Nicotinic acid (B3)	0.05
Pantothenic acid (B5)	0.05
Cyanocobalamin (B12)	0.002
4-aminobenzoic acid (B10)	0.05

Table S2 – Description of analytical methods employed for the quantification of toluene, acetate, H_2, CH_4, O_2

**Toluene analysis**	
GC system	Agilent 8860 with FID detector
Column	Agilent DB-624 GC fused silica (60m x 0.53 mm inner diameter, 0.3 μm thickness)
Operational parameters	GC method: Carrier gas: nitrogen 7 mL/min; Injection Temp: 250 °C; Interface Temp: 300 °C; Oven Temp Program: 100°C for 1.0 min, then ramp 20°C/min up to 150°C, hold for 1 min
Calibration range	0 – 29 ppm
LOD	0.3 ppm

LOQ	1 ppm		
**Acetate analysis**			
GC system	Agilent 8860 with FID detector		
Column	Agilent DB-FFAP GC fused silica (30m x 0.53 mm inner diameter, 1.5 μm thickness)		
Operational parameters	GC method: Carrier gas: nitrogen 8.7 mL/min; Injection Temp: 250 °C; Oven Temp Program: 110°C for 1.0 min, then ramp 10°C/min up to 150°C, then ramp 20°C/min up to 175°C, hold for 2 min		
Calibration range	0 – 150 ppm		
LOD	0.3 ppm		
LOQ	1 ppm		
**Gas analysis**			
GC system	Agilent 8860 with TCD detector		
Column	Agilent Carboxen 1000 stainless steel packed (3.05 m x 0.32cm, OD, 2 mm ID, Carboxen-1000 packing, mesh size 60/80, pre-conditioned)		
Operational parameters	GC method: Carrier gas: Nitrogen 10 mL/min; Injection Temp: 200 °C; Interface Temp: 300 °C; Oven Temp Program: 100°C for 10.0 min		
Gas	H_2	CH_4	O_2
Calibration range (ppm)	23 – 69	1.5 – 6.1	65 - 392
LOD	0.3 ppm	0.3 ppm	0.3 ppm
LOQ	1 ppm	1 ppm	1 ppm

Sulfate reduction inhibition experiment

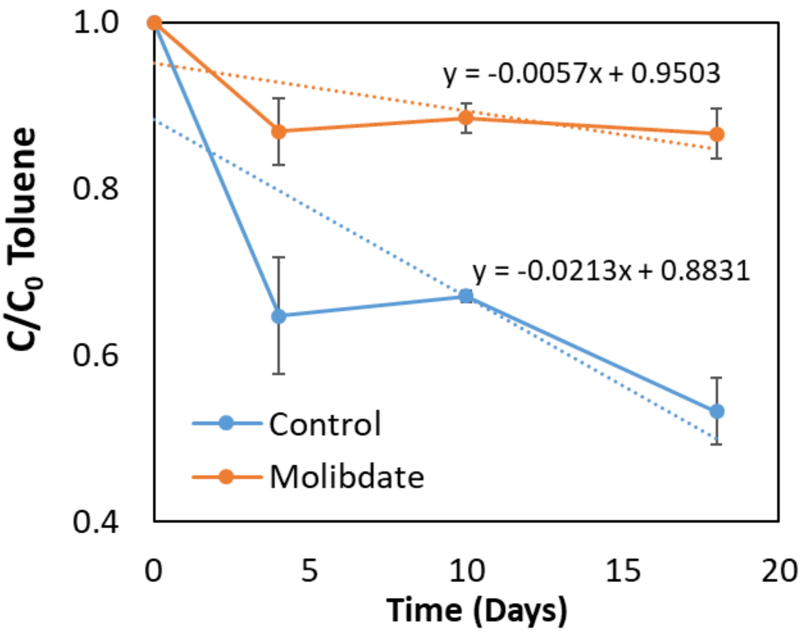

FigureS1 - Trends of toluene consumption in presence and absence of molibdate 20Mm in microcosm experiments containing excess of sulfate and inoculated with the original enriched culture.
